# Profiling of spatial metabolite distributions in wheat leaves under normal and nitrate limiting conditions

**DOI:** 10.1016/j.phytochem.2015.01.007

**Published:** 2015-07

**Authors:** J. William Allwood, Surya Chandra, Yun Xu, Warwick B. Dunn, Elon Correa, Laura Hopkins, Royston Goodacre, Alyson K. Tobin, Caroline G. Bowsher

**Affiliations:** a School of Chemistry, Manchester Institute of Biotechnology, 131 Princess Street, Manchester M1 7DN, UK; b Faculty of Life Sciences, University of Manchester, Michael Smith Building, Oxford Road, Manchester M13 9PL, UK; c Manchester Centre for Integrative Systems Biology, Manchester Institute of Biotechnology, 131 Princess Street, Manchester M1 7DN, UK; d Centre for Advanced Discovery and Experimental Therapeutics (CADET), Central Manchester University Hospitals NHS Foundation Trust, York Place, Oxford Road, Manchester M13 9WL, UK; e School of Biology, Biomolecular Sciences Building, University of St Andrews, St Andrews, Fife, KY16 9ST Scotland, UK; f Vice Chancellor’s Office, York St John University, Lord Mayor’s Walk, York YO31 7EX, UK; g School of Biosciences, University of Birmingham, Edgbaston, Birmingham B15 2TT, UK

**Keywords:** *Triticum aestivum*, Wheat, Leaves, Metabolite fingerprinting, Metabolite profiling, Bayesian network analysis, Nitrate

## Abstract

The control and interaction between nitrogen and carbon assimilatory pathways is essential in both photosynthetic and non-photosynthetic tissue in order to support metabolic processes without compromising growth. Physiological differences between the basal and mature region of wheat (*Triticum aestivum*) primary leaves confirmed that there was a change from heterotrophic to autotrophic metabolism. Fourier Transform Infrared (FT-IR) spectroscopy confirmed the suitability and phenotypic reproducibility of the leaf growth conditions. Principal Component–Discriminant Function Analysis (PC–DFA) revealed distinct clustering between base, and tip sections of the developing wheat leaf, and from plants grown in the presence or absence of nitrate. Gas Chromatography-Time of Flight/Mass Spectrometry (GC-TOF/MS) combined with multivariate and univariate analyses, and Bayesian network (BN) analysis, distinguished different tissues and confirmed the physiological switch from high rates of respiration to photosynthesis along the leaf. The operation of nitrogen metabolism impacted on the levels and distribution of amino acids, organic acids and carbohydrates within the wheat leaf. In plants grown in the presence of nitrate there was reduced levels of a number of sugar metabolites in the leaf base and an increase in maltose levels, possibly reflecting an increase in starch turnover. The value of using this combined metabolomics analysis for further functional investigations in the future are discussed.

## Highlights

• Nitrogen and carbon assimilation interaction is essential in leaf metabolism.
• Along the developing wheat leaf there is a switch from hetero- to auto-trophy.
• GC–MS metabolite profiling was combined with Bayesian network (BN) correlation analysis.
• Amino acid, organic acid and carbohydrate distribution changed with nitrate levels.

## Graphical abstract

Classical phenotyping and metabolite profiling were combined to study the spatial development of photosynthetic capacity within the developing wheat leaf, revealing *bonafide* metabolic changes associated with hetero- and auto-trophy.

**Figure:**
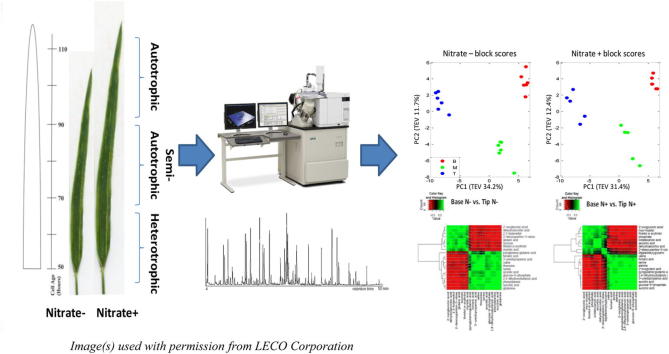

## 1 Introduction

Nitrogen is a major plant nutrient, being an essential component of amino acids, peptides and proteins, chlorophyll, nucleic acids and many cofactors and plant defence compounds. For most higher plants, particularly when growing in well-aerated soils, nitrate is the primary source of inorganic nitrogen. Nitrate is reduced to nitrite, then ammonium, prior to assimilation into amino acids, in a series of reactions that are highly compartmentalised within cells and tissues (Tobin and Yamaya, 2001). Nitrogen assimilation interacts with carbon assimilation and degradation in a complex network that adjusts the balance between N and C according to the physiological status of the tissue and the environmental conditions (Nunes-Nesi et al., 2010), in both photosynthetic and non-photosynthetic tissue (Smirnoff and Stewart, 1985).

Nitrate assimilation and amino acid biosynthesis require a supply of reductant (NAD(P)H and/or reduced ferredoxin) and ATP as well as a range of organic acids to act as carbon skeletons. In photosynthetic cells, reductant and ATP can be derived from photosynthesis, while mitochondrial respiration also provides supplementary ATP and reductant even in light (Kromer, 1995; Nunes-Nesi et al., 2010). Carbon skeletons can be produced from newly synthesised carbohydrates that are converted into organic acids via respiration (glycolysis, TCA cycle and oxidative pentose phosphate pathway), or from stored organic compounds (Nunes-Nesi et al., 2010; Sweetlove et al., 2010). In non-photosynthetic cells, reductant and ATP are supplied by respiration, with carbohydrates being imported, as sucrose from photosynthetic tissue or released from reserves. The cost of transporting sucrose to the roots, as well as respiration to generate ATP and reductant, makes roots and other non-photosynthetic tissue more energetically ‘costly’ as sites for nitrate assimilation. The energetic advantages of photosynthetic tissue becomes increasingly compromised as light intensities fall to the point where photosynthesis becomes light-limited. Under these conditions nitrate assimilation competes with the Calvin cycle for reductant and ATP, leading to a reduced rate of carbon assimilation (Canvin and Atkins, 1974). If carbohydrate concentrations fall it can result in a depletion of amino acid pools, either due to a limited supply of carbon skeletons for amino acid synthesis or due to the catabolism of amino acids to maintain respiration (Matt et al., 1998; Usadel et al., 2008). Even under high light, the presence of nitrate can shift the flow of photosynthetic carbon towards amino acid (Champigny and Foyer, 1992) and organic acid synthesis (Scheible et al., 1997), while carbohydrate synthesis is decreased, and a greater proportion of assimilated carbon is incorporated into organic and amino acids (Stitt et al., 2002). These examples illustrate the need for nitrogen and carbon assimilation pathways to be coordinated in order that there is an adequate supply of carbon to support amino acid biosynthesis without compromising growth. They also indicate significant differences in the metabolic networks that exist within non-photosynthetic compared to photosynthetic tissue.

To date there have been some extremely informative integrative ‘omics’ approaches for assessing nitrogen status in the model dicotyledonous plant Arabidopsis. For example Hirai et al. (2004) combined transcriptomics and metabolomics to gain a better understanding of nutritional stress responses in Arabidopsis. Whilst Albinsky et al. (2010) over expressed rice full-length cDNA clones in Arabidopsis and then performed transcriptome and metabolome analyses to learn more about the processes related to nitrogen metabolism. A more sophisticated experimental design and the measurement of relevant enzyme activities, in addition to classical targeted metabolite quantification, allowed Tschoep et al. (2009) to interpret Arabidopsis nitrogen deficiency phenotypes. Such studies in Arabidopsis have, by necessity, not accounted for the fact that certain tissues of the leaf are undergoing different metabolic processes with respect to autotrophic and heterotrophic metabolism. Also all these studies have used multiple leaf pools from multiple plants meaning that it is not possible to compare respiring versus photosynthetic tissues and no consideration can be given to leaves from different positions and of different ages.

In order to identify metabolic networks and their fluctuations in response to changing N supply and C assimilation, we have used the natural developmental gradient that exists within the wheat primary leaf. This system has advantages over comparisons between leaf and root assimilation because it provides tissue that is anatomically comparable (i.e. composed of mesophyll, vascular and epidermal cells) and is readily characterised. As cell division is restricted to a basal meristem, this generates a measurable gradient of cell age and development along the leaf blade with a transition from non-photosynthetic cells at the base to fully photosynthetic cells at the leaf tip (Tobin et al., 1985). Hence, within a single tissue we are able to identify distinct changes in metabolic networks as the pathways for nitrogen assimilation operate within cells that are transitioning from wholly respiratory to fully photosynthetic.

In this paper initial studies were carried out to characterise the physiological differences between basal and mature regions of wheat primary leaves of nitrate-grown plants. Following characterisation by metabolite fingerprinting with Fourier Transform Infrared (FT-IR) spectroscopy, non-photosynthetic, semi-autotrophic and fully photosynthetic leaf sections were taken from plants grown in the presence or absence of nitrate and subjected to metabolite profiling using Gas Chromatography-Time of Flight/Mass Spectrometry (GC-TOF/MS). The metabolite data were analysed using multivariate chemometric approaches, point-by-point (univariate) data interpretation, as well as by Bayesian network (BN) based correlation analyses.

We discuss the extent to which these metabolomics approaches are able to distinguish the different tissues and treatments and we identify major changes in metabolite networks during the transition from heterotrophic to fully photosynthetic metabolism in response to increased N supply. The value of this approach when undertaking functional investigations of plants grown in different scenarios is also considered.

## 2 Results

### 2.1 Changes in metabolism during leaf development

**Fig. 1:**
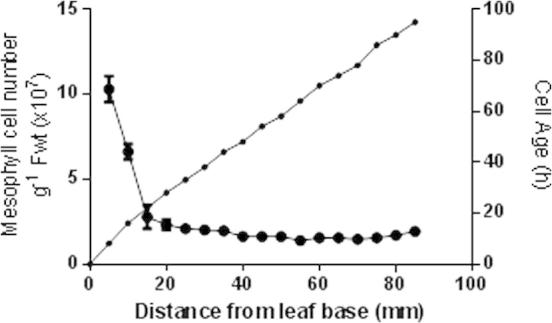
Mesophyll cell number and age along the developing wheat leaf. Mesophyll cell numbers (large closed circle) and cell age (small closed circle) along the length of 7 day old primary leaves. Data points represent the mean of 5 independent growth studies, sampling 5 seedlings per replicate. Error bars show ±SE of the mean.

In wheat plants grown in the presence of a continuous N supply by growing on compost, mesophyll cell number was highest in the basal 5 mm of the leaf blade (approximately 11 × 10^7^ cells g^−^^1^ fwt), and then rapidly decreased to a constant lower number of ca. 2 × 10^7^ cells g^−^^1^ fwt beyond 20 mm from the leaf base (Fig. 1), confirming that cell division is restricted to the basal 5 mm and the mesophyll cell elongation zone occurred within the basal 20 mm of the leaf (0–20 h old). Cell age increases rapidly over the basal region, where the cells are actively dividing, and then increases at a constant rate with distance from the leaf base (Fig. 1).

**Fig. 2:**
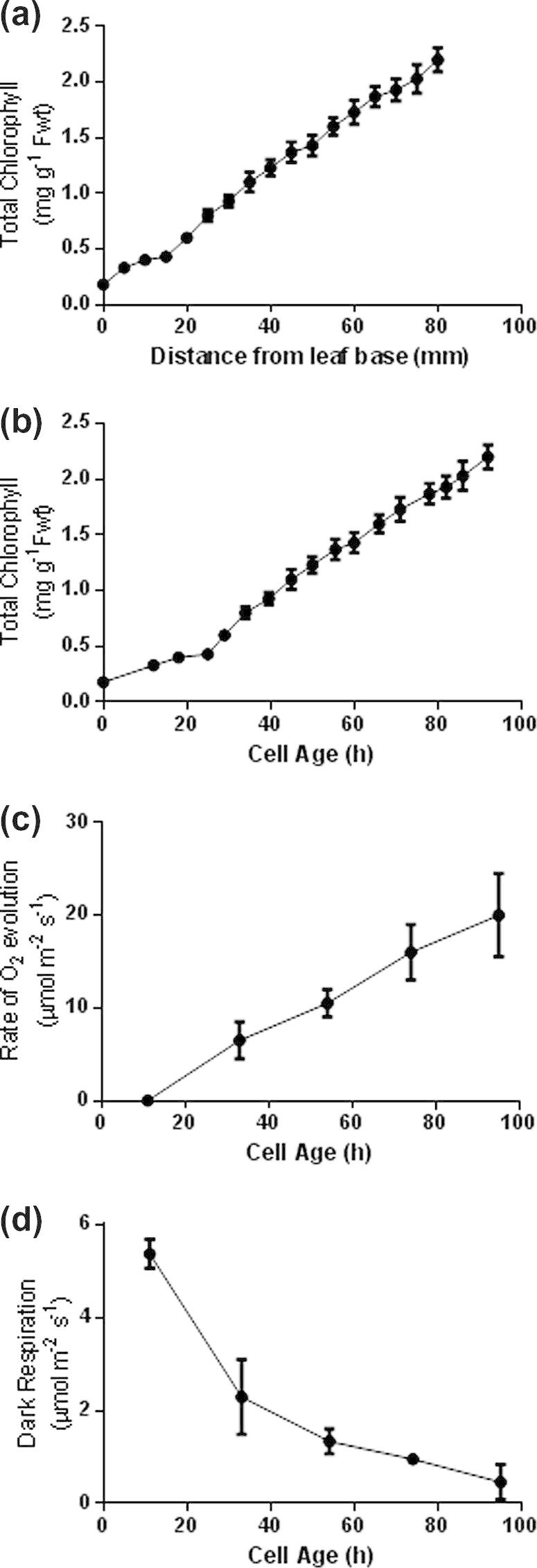
Changing chlorophyll concentration, CO_2_ dependent O_2_ evolution and dark respiration along the length of 7 day old primary wheat leaves. Chlorophyll concentration was measured on a (a) spatial, and (b) temporal scale. (c) CO_2_ dependent O_2_ evolution was measured using a leaf disc electrode at saturating concentration of CO_2_. (d) Dark respiration was also measured using a leaf disc electrode. Data points represent the mean of at least 3 independent growth studies, sampling at least 5 seedlings per replicate. Error bars show ±SE of the mean.

**Fig. 3:**
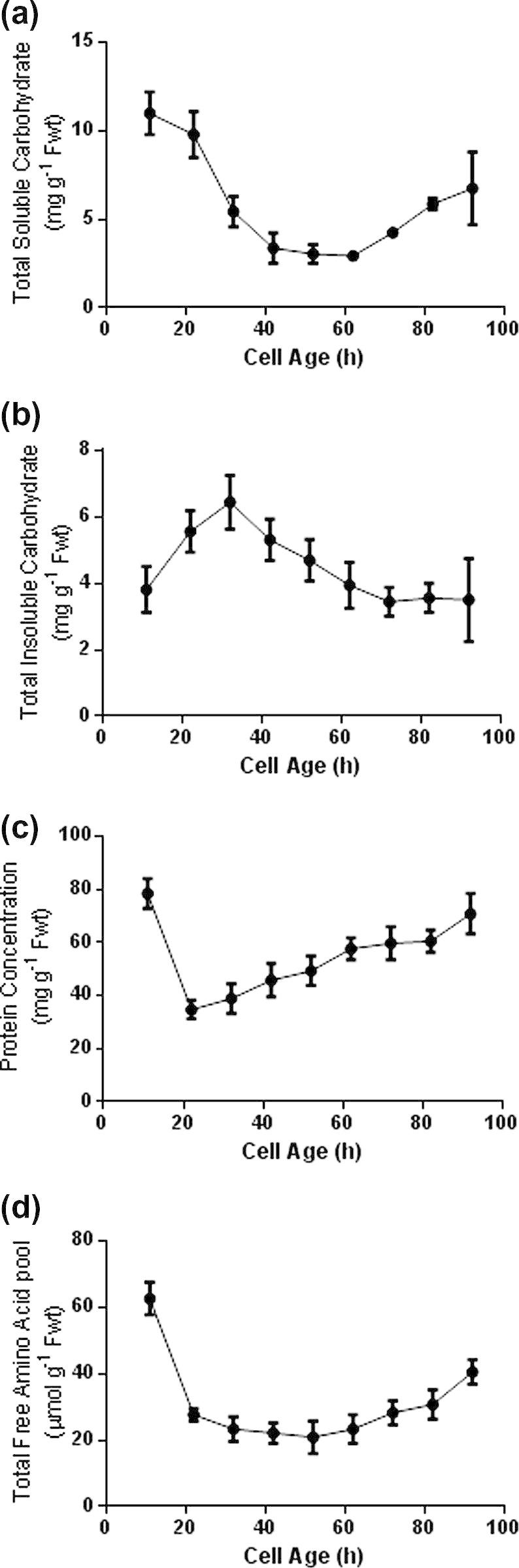
Soluble and insoluble carbohydrate, protein and free amino acid, changes along the developing wheat leaf. Changes in (a) soluble carbohydrate, (b) insoluble carbohydrate, (c) protein, and (d) total free amino acid pool in relation to cell age along the length of 7 day old primary wheat leaves. Data points represent the mean of a minimum of 5 independent growth studies, sampling at least 5 seedlings per replicate. Error bars show ±SE of the mean.

The total chlorophyll concentration markedly increased from the leaf base to the tip (Fig. 2a and b). The data are plotted in alternative forms to show how distance along the leaf from the base (Fig. 2a) equates to cell age in hours (Fig. 2b). In subsequent graphs the data are presented against cell age. There is a ‘switch over’ from heterotrophic metabolism, where respiration predominates up until the end of the elongation zone of the leaf (Fig. 2d), to autotrophic metabolism, where photosynthesis predominates towards the leaf tip (Fig. 2c). Photosynthetic activity reaches its maximum at the leaf tip, coinciding with the maximum size and development of the chloroplasts (Figs. 2c, S1). Based on this metabolic distinction, metabolite fingerprinting and profiling of the basal, mid and terminal 20 mm sections of the developing wheat leaf allows a comparison to be made between heterotrophic, ‘semi-autotrophic’ and fully autotrophic metabolism. Basal tissue contains cells up to 24 h old, which includes all the meristematic cells as well as those undergoing elongation. Although they contain some chlorophyll (Fig. 2b) there is no detectable photosynthesis (Fig. 2c) and they are dependent on respiration (Fig. 2d) to supply ATP and reductant for nitrogen assimilation. Carbohydrates are present, with the soluble forms predominating (Fig. 3a and b). The soluble protein concentration (Fig. 3c) showed maximal levels in the youngest cells at the leaf base, rapidly decreasing to a minimum towards the end of the elongation zone (20 h). In the mid-section (60–80 mm from the base) the cells are 70–90 h old and while respiratory activity has decreased (Fig. 2d) they are still developing photosynthetically, reaching 50% of the maximum capacity that is attained at the leaf tip (Fig. 2c). In this region of the leaf the soluble carbohydrate (Fig. 3a), proteins (Fig. 3c) and amino acid pools (Fig. 3d) are beginning to increase. Finally, the tip sections are fully developed, with minimum rates of dark respiration (Fig. 2d) and maximum rates of photosynthesis (Fig. 2c).

### 2.2 Metabolite fingerprinting of wheat leaf development

**Fig. 4:**
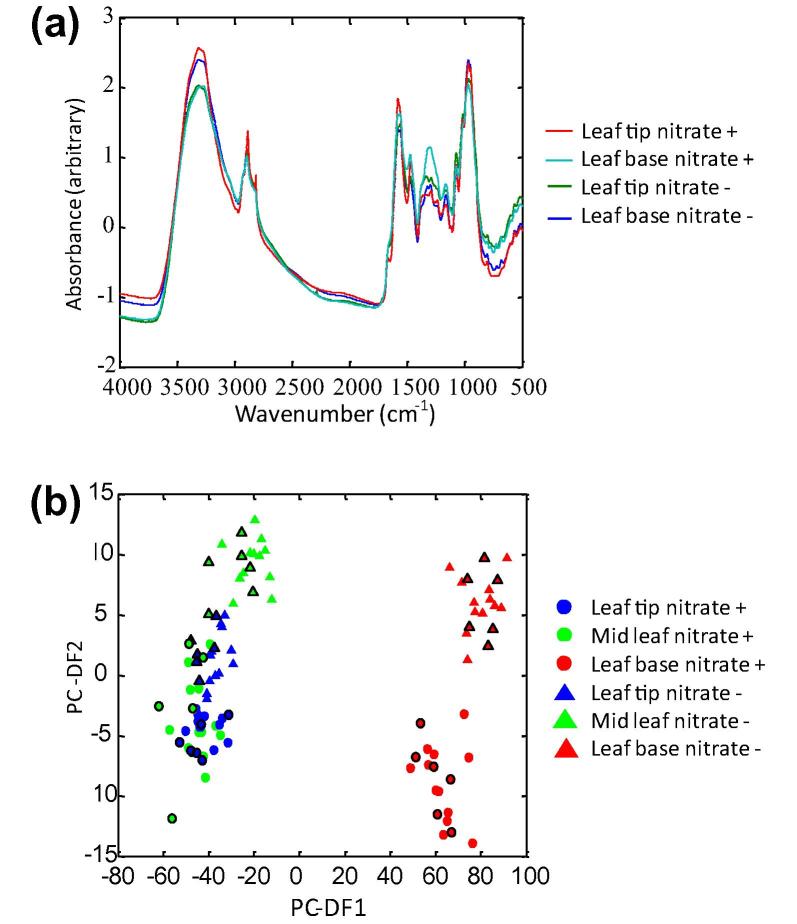
FT-IR spectra and data analysis of primary wheat leaf sections from plants grown in the presence or absence of nitrate. FT-IR analysis of primary wheat leaf base, mid and tip sections taken from plants grown in the presence or absence of nitrate. (a) SNV baseline corrected FT-IR absorbance spectra. (b) PC–DFA scores plot of FT-IR spectra. The validated PC–DFA model was based on independent projection of a test dataset (black border on symbol) on to a training dataset model. The data represents six biological replicates analysed in triplicate per class (*n* = 18). Leaf base in presence (red circle) or absence (red triangle) of nitrate, mid leaf in presence (green circle) or absence (green triangle) of nitrate, leaf tip in presence (blue circle) or absence (blue triangle) of nitrate.

FT-IR spectroscopy was first used to assess the suitability and reproducibility of the leaf system growth conditions, as well as sample harvest and enzymatic quenching protocols. It also provided a rapid fingerprint (or phenotypic) comparison of the biochemical composition for each experimental class. A Standard Normal Variate (SNV) baseline correction was performed on the FT-IR spectra (Fig. 4a), which was then followed by the calculation of the first derivative spectra. PC–DFA was generally capable of discriminating the experimental classes (base, mid and tip) taken from plants grown in the presence or absence of nitrate (Fig. 4b). The co-clustering of the test and the training data indicates a high degree of reproducibility between biological and analytical replicate data of the same class (Fig. 4b), suggesting that the experimental approach was appropriate for more in depth metabolite profiling.

### 2.3 GC-TOF/MS metabolite profiling of nitrate supplemented and limited developing wheat leaves

**Fig. 5:**
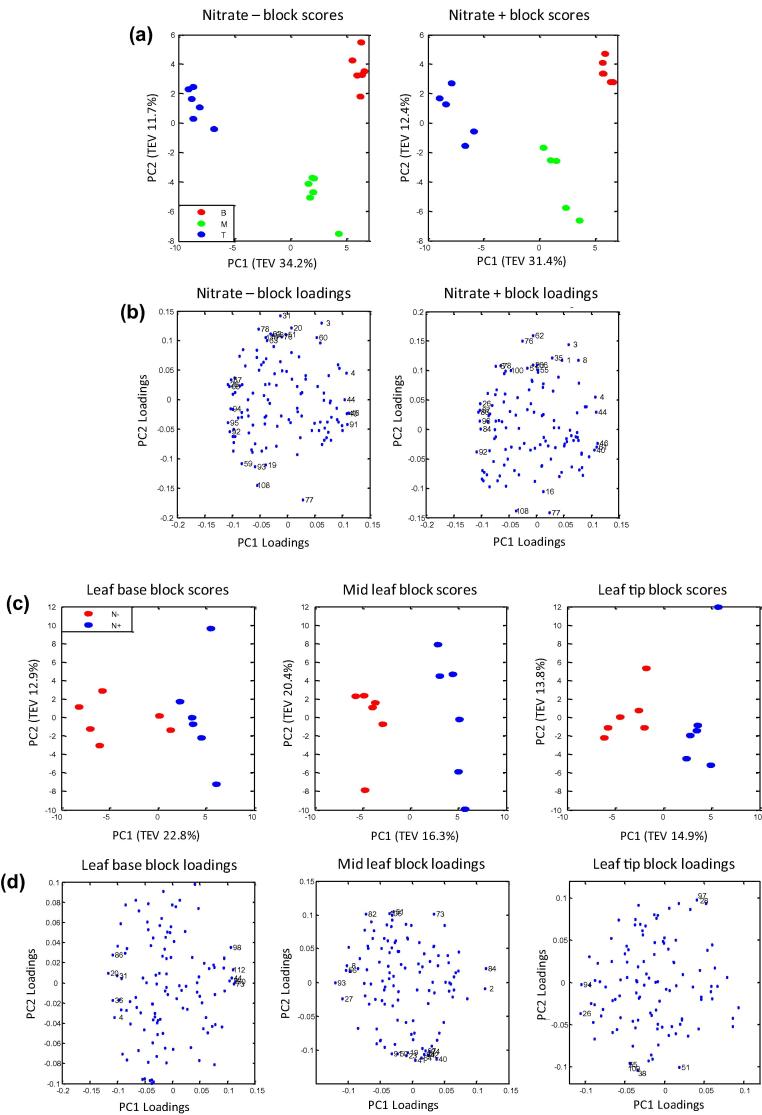
Multiblock C-PCA of GC-TOF/MS metabolite profiles. (a) C-PCA scores plot (six biological replicates per class), the data were divided into two blocks corresponding to presence (+) and absence (−) of nitrate; the total explained variance (TEV) for each PC is given. Leaf base (B red circle), mid leaf (M green circle) and leaf tip (T blue circle). (b) C-PCA loadings plot corresponding to (a). Variables with loadings scores within the ±0.1 threshold are labelled, index numbers correspond to Table S1. (c) C-PCA scores plot based upon six biological replicates for each of the six experimental classes, the data were divided into three blocks corresponding to leaf base, mid leaf, and leaf tip sections. Labels indicate plants grown in the presence (N+ blue circle) and absence (N− red circle) of nitrate. (d) C-PCA loadings plot corresponding to (c). Variables with loadings scores within the ±0.1 threshold are labelled, index numbers correspond to Table S1.

A total of 115 metabolite features were detected by GC-TOF/MS in wheat primary leaves grown in the presence or absence of nitrate, of which a total of 51 metabolites were identified by library matching (Table S1). Chemometric analysis of the GC-TOF/MS metabolite profiles focused upon the selection of differentially expressed metabolites that revealed significant trends either between the leaf regions or in response to growing the plant in the presence or absence of nitrate. Three approaches to the data mining were applied. First multiblock Consensus (C)-PCA was applied (Biais et al., 2009; Smilde et al., 2003; Westerhuis et al., 1998; Xu and Goodacre, 2012), where models combine several different but potentially connected data sets (called “blocks”), with emphasis upon modelling the “common trend” between the blocks. The sample distribution of each individual block are shown in their respective “block scores” plot and the contribution of metabolites in relation to the observed trend are shown in their “block loadings” plot (Biais et al., 2009). The first C-PCA model (Fig. 5a and b) arranged the data into two blocks consisting of nitrate supplemented and nitrate deprived samples. The second C-PCA model (Fig. 5c and d) arranged the data into three blocks consisting of leaf base, mid leaf, and leaf tip. The multiblock C-PCA scores plot (Fig. 5a) gave distinct clustering patterns for all three leaf regions within the two blocks corresponding to the presence or absence of nitrate, the multiblock C-PCA scores plot (Fig. 5c) also gave distinct clustering patterns for plants grown in the presence or absence of nitrate within the three blocks corresponding to each leaf section, and thus the respective PC loadings were derived (Fig. 5b and d) and further investigated. Secondly variable selection analyses using the univariate Wilcoxon rank-sum test were performed (Table S1). Each of the three leaf tissue sections were compared under the two respective nitrate conditions, and each respective tissue section was compared between the two nitrate conditions. Finally, BN analyses were performed upon all features where a metabolite identification was attained via library matching and focused upon comparisons of the leaf base (fully heterotrophic) and tip (fully autotrophic) in the absence or presence of nitrate.

### 2.4 Metabolite levels altered during wheat leaf development

**Fig. 6:**
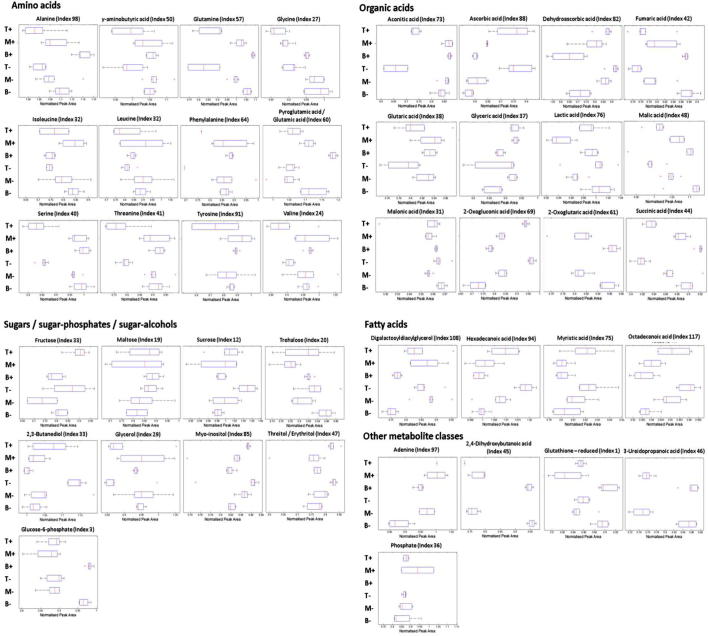
Box and whisker trend plots of significant metabolites determined by GC-TOF/MS. Metabolite levels in leaf base (B), mid leaf (M) and leaf tip (T) sections from the developing primary wheat leaves of plants grown in the presence (+) or absence (−) of nitrate measured using GC-TOF/MS. The normalised peak areas representing the mean of each metabolite and experimental class are displayed as box and whisker plots. Within the plots, the box represents the interquartile range (25% and 75%), the whiskers (error bars) represent data points not considered as outliers defined by 1.5× the interquartile range deviation from the mean. In cases where the data distribution is very tight and values lie only just outside of the 25% or 75%, the whiskers may not be clearly visible due to overlap with the box.

**Fig. 7:**
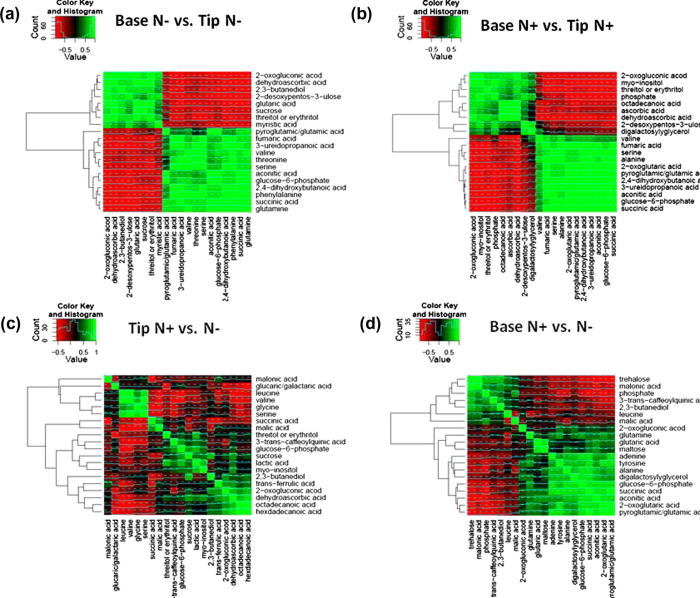
Bayesian network (BN) analysis correlation heat maps. Bayesian network (BN) analysis was performed to search for strong probabilistic correlations between metabolites with respect to growth conditions and leaf position. The BN results were translated into Pearson’s correlation coefficients and are displayed as a heat map. Strong correlations were searched between metabolites within (a) the base and tip of the leaf when grown in the absence of nitrate; (b) the base and tip of the leaf when grown in the presence of nitrate; (c) the leaf tip when grown in the absence and presence of nitrate; (d) the leaf base when grown in the absence and presence of nitrate.

Of the metabolites with known identity, 35 (68.6%) were differentially present between at least one of the leaf regions on the basis of the Wilcoxon rank-sum test (False Discovery Rate (FDR) *q*-value 0.05) (Fig. 6, Table S1). The results of BN analysis are presented in the form of correlation heat maps (Fig. 7), a traditional network topology (Fig. S2) and plots of peak area log_2_ ratio (Fig. S3). After BN analysis, *t*-tests (FDR *q*-value 0.05) are applied to highlight the metabolites that are significantly altered. The significant metabolites are displayed as traditional correlation networks (Fig. S2a–h). To simplify the presented results, each network is split into two component parts. For example, all metabolites which were significantly altered in the base or tip regions of plants grown in the absence of nitrate are correlated in a network based on the base (Fig. S2a) or tip (Fig. S2b) data only. As a first step BN analysis was used to assess metabolites that strongly correlated between the leaf base and tip when grown in the absence (Figs. 7a, S2a,b, S3a,b) or presence (Figs. 7b, S2c,d, S3c,d) of nitrate.

The levels of the amino acids, gamma-aminobutyric acid (GABA), alanine, glutamine, glutamic acid, glycine, leucine, phenylalanine, serine, threonine, tyrosine and valine decreased significantly between the base and the tip of the leaf (Fig. 6, Table S1). These changes in amino acids were confirmed significant with both the Wilcoxon rank-sum test and BN analyses (Table S1, Figs. 7a and b, S2a–d, S3a–d), indicating the approaches were significantly complementary.

BN analysis and application of the Wilcoxon rank-sum test both indicated that levels of TCA cycle organic acids, including fumaric-, malic-, aconitic- and succinic-acids significantly decreased between the leaf base and tip sections (Figs. 6, 7a and b, S2a–d, S3a–d, Table S1). By contrast, the levels of dehydroascorbic and 2-oxoglutaric acids significantly increased from the base to the mid and to the tip of the leaf (Figs. 6, 7a and b, S2a–d, S3a–d, Table S1). Lactic acid was decreased in the mid leaf compared to the tip and base under both nitrate conditions (Fig. 6, Table S1).

The level of sucrose increased significantly from the base to the mid and tip of the leaf, as revealed by Wilcoxon rank-sum test and BN analyses. Other unidentified sugar metabolites (monosaccharides and/or disaccharides and/or sugar alcohols) 7, 53, 59, 90, 92, 93, and 95 (Table S1) increased significantly from the base to the tip section of the leaf based upon multiblock C-PCA loadings (Fig. 5a and b, Table S1) and the Wilcoxon rank-sum test (Table S1). Multiblock C-PCA and BN analysis indicated that maltose and fructose levels were significantly greater within the leaf tip (Figs. 5a and b, 7a and b, S2a–d, S3a–d, Table S1). In contrast, the level of glucose-6-phosphate (G-6-P) declined significantly between the leaf base and mid leaf, but remained constant to the leaf tip, as indicated by all three statistical approaches (Figs. 5a and b, 6, 7a and b, S2a–d, S3a–d, Table S1).

The levels of sugar alcohols, including 2,3-butanediol, threitol and/or erythritol and myo-inositol, increased significantly in the leaf tip compared to the base (Fig. 6, Table S1). Similarly, the Wilcoxon rank-sum test and BN analysis indicated that the levels of the fatty acids, octadecanoic-, myristic- and hexadecanoic-acid, increased significantly along the length of the leaf from base to tip. In contrast, 2,4-dihydroxybutanoic acid and 3-ureidopropanoic acid, decreased significantly from the leaf base to the tip (Figs. 6, 7a and b, S2a–d, S3a–d, Table S1).

### 2.5 Comparison of differential metabolite profiles of each leaf region in response to nitrate supplementation

To assess which metabolites altered between nitrate conditions for each respective leaf section, C-PCA loadings were first derived (Fig. 5, Table S1) and the Wilcoxon rank-sum test was applied, where the null hypothesis was that no significant difference was observed between the metabolite levels of the respective leaf sections when plants were grown in the presence compared to the absence of nitrate (Table S1). BN analyses were in this case applied to compare the leaf tip (Figs. 7c, S2e,f, S3e,f), or the base (Figs. 7d, S2g,h, S3g,h) when grown in the presence or absence of nitrate.

Multiblock C-PCA and BN analysis indicated that the level of maltose within the leaf base was significantly higher in nitrate supplemented plants (Figs. 5c and d, 6, 7d, S2g, S3g). In contrast, significantly lower levels of trehalose were detected in the basal region of leaves in nitrate supplemented plants, as indicated by all three statistical approaches (Figs. 5c and d, 6, 7d, S2h, S3h).

Amino acid levels showed differential responses in sections taken from the primary leaf of plants grown in the presence or absence of nitrate, as indicated by Wilcoxon rank-sum test and BN analysis (Figs. 6 and 7, Table S1). The levels of leucine in the basal region of leaves were significantly lower in nitrate supplemented plants (Figs. 6, 7d, S2h, S3h, Table S1). In contrast, the level of glutamic acid, tyrosine, GABA and alanine were significantly higher in the basal region of leaves in nitrate supplemented plants (Figs. 6, 7d, S2g, S3g, Table S1).

As confirmed by both the Wilcoxon rank-sum test and BN analysis, the levels of the organic acids aconitic- and succinic-acid increased significantly in the leaf base region in nitrate supplemented plants (Figs. 6, 7d, S2g, S3g, Table S1). By contrast, a significant decrease in the levels of 2-oxoglutaric- and dehydroascorbic-acid were observed in the tip section of nitrate supplemented plants (Figs. 6, 7c, S2e,f, S3e,f).

Further metabolites of interest that were mined by BN analysis (Figs. 7d, S2h, S3h, Table S1) included, sucrose, lactic acid, hexadecanoic acid, and 2,3-butanediol, which were all of greater concentration in the leaf base of plants grown in the absence of nitrate (Fig. 6).

## 3 Discussion

### 3.1 Metabolic changes

The developing primary wheat leaf provides a model system in which a gradient of cells exists along the leaf blade in terms of both age and development (Boffey et al., 1979; Ellis et al., 1983; Leech, 1985; Tobin et al., 1985). Our metabolic studies on wheat plants grown on compost with a continuous supply of nitrogen confirmed that photosynthesis, dark respiration and carbohydrate metabolism all changed with development in a manner comparable to that previously reported for *Triticum aestivum* var Maris Huntsman (Baker and Leech, 1977), *Zea mays* (Baker and Leech, 1977; Ponnala et al., 2014), *Fescue arundinacea* (Allard and Nelson, 1991) and *Hordeum vulgare* (Bowsher and Tobin, 2001; Thompson et al., 1998). High rates of net deposition of proteins, soluble carbohydrates and amino acids occurred in the youngest cells while the highest net rate of deposition of insoluble carbohydrates (as structural or storage reserves) occurred within the zone of elongation. Such changes reflect a change in metabolism or mobilisation due to the heterotrophic or autotrophic nature of the cells present at different positions within the leaf blade. For example, in young non-photosynthetic cells at the leaf base, the soluble carbohydrates will have been transported from the photosynthetic cells at the leaf tip primarily in the form of sucrose (Allard and Nelson, 1991).

### 3.2 Metabolite fingerprinting and profiling

Metabolic differences were confirmed by FT-IR spectroscopy, with distinct clusters between the base and tip leaf sections from plants grown in the presence or absence of nitrate being observed in the PC–DFA scores plot. In contrast the mid-leaf section was less tightly clustered reflecting the semi-autotrophic nature of metabolism in this tissue. This combination of FT-IR spectroscopy with appropriate chemometrics has previously been successfully employed in the classification of olive oil (Lai et al., 1994), adulteration of cocoa butters (Goodacre and Anklam, 2001), for plant breeding (Goodacre et al., 2007a), the examination of salinity effects on tomato fruit (Johnson et al., 2003), to investigate susceptible and resistant interactions of the model plant *Brachypodium distachyon* with the rice blast pathogen *Magnaporthe grisea* (Allwood et al., 2006), the response of *Arabidopsis thaliana* to *Pseudomonas syringae* pv. *tomato* (Allwood et al., 2010), and in identifying biochemical variations in heather leaf tissue in response to nitrogen level (Gidman et al., 2003, 2005). Applying FT-IR fingerprinting as reported here in a well-characterised monocot model, can be used to identify the parts of the plant that have the strongest differential response to a varied N-supply. The high levels of sample throughput provided by FT-IR can permit screening large ranges of plant genotypes and nutrient conditions in high replication, prior to selecting the most informative nutrient condition groups and genotypes to apply more in depth and higher expense GC–MS profiling to.

The GC-TOF/MS profiles were analysed by multiblock C-PCA revealing distinct clustering between leaf sections of plants grown in the presence or absence of nitrate (Fig. 5a and b), and between respective tissue sections from plants grown in the presence or absence of nitrate (Fig. 5c and d). Of 115 metabolites detected, 39 were of known chemical structure as defined by matching the retention index and electron impact mass spectrum to authentic chemical standards measured in-house (Level 1 identification; Table S1; Sumner et al., 2007), a further 12 were of level 2 identification (Table S1) due to MS matching to external metabolite libraries alone (NIST/EPA/NIH05 and Golm Metabolome Database; Hummel et al., 2010; Kopka et al., 2005; Sumner et al., 2007). Thirty-five metabolites of known identity showed significant changes between the leaf regions when assessed at the univariate level by the Wilcoxon rank-sum test (Table S1).

BN analysis is a multivariate statistical technique that has only recently been applied to metabolomics investigations (Gavai et al., 2009; Li and Chan, 2004). A BN is a graphical model of a probability distribution over a set of variables of a given problem domain (Jensen, 2001; Neapolitan, 2003), providing a compact and intuitive representation of their relationships. These relationships or correlations are broadly either (a) “positive correlations” which indicate that the values of both variables increase or decrease together, and (b) “negative correlation” which indicates that as one variable increases, the other decreases. The network structure of a BN encodes probabilistic dependencies among domain variables and a joint probability distribution quantifies the strength of these dependencies (Heckerman, 1995). The resulting graphical model (network) allows (i) *Visualisation of probabilistic relationships*: providing direct information on underlying interactions between metabolites, and (ii) *Inference*: where the BN is used to predict outputs or to classify new samples (Correa and Goodacre, 2011).

The trends of significant metabolite levels during leaf development and in response to nitrate were mined using this combination of multivariate based cluster analyses (C-PCA), point-by-point univariate analyses (Wilcoxon rank-sum test), and BN correlation analysis, and then are most easily visualised using the box and whisker plots based on experimental class averages (Fig. 6).

The rate of respiration was highest in the youngest tissue at the base of the primary leaf, and decreased as the leaf became more photosynthetically developed. Consistent with earlier observations made by Tobin et al. (1988), the level of the TCA cycle compounds fumaric-, malic-, 2-oxoglutaric- and succinic-acids, decreased from the base to the tip of the leaf. As substrates for the four enzymes identified as the major controlling points in the cycle, namely, fumarase, malate dehydrogenase, 2 oxoglutarate dehydrogenase and succinate dehydrogenase (Araujo et al., 2012), the accumulation of these metabolites is indicative of a high flux through the TCA cycle in the young cells at the leaf base. Furthermore, the levels of the respiratory metabolite aconitic acid is correlated with carbohydrates which support respiration (Figs. S2a, S3a). Fumaric acid is a key metabolite in the base and is negatively correlated to TCA metabolites and carbohydrates.

The amino acids alanine, glutamic acid, glutamine and threonine were significantly higher in the base than the tip of the primary leaf. The extent and rate of accumulation of different amino acids markedly varies depending on the plant species and growth conditions (Fritz et al., 2006; Gibon et al., 2006; Leidreiter et al., 1995; Lohaus et al., 1995; Noctor et al., 2002; Nunes-Nesi et al., 2010; Scheible et al., 1997). Such changes reflect the rate of accumulation and the rate at which amino acids are used for protein synthesis and their rate of export in the phloem. Succinic acid was correlated with a number of aromatic amino acids, suggesting a role as the source of C skeletons.

The levels of sucrose, maltose, fructose and a number of unidentified sugar metabolites increased towards the leaf tip, as photosynthetic activity developed. Similarly, metabolite profiling identified an increasing fraction of photosynthate maintained as sucrose as the developing quaking aspen leaf expands (Jeong et al., 2004). This will provide a source of carbohydrates to the young cells at the leaf base (Allard and Nelson, 1991; Sweetlove et al., 2010).

Fatty acid synthesis in leaves takes place mostly in the chloroplast (Harwood, 1975) and requires cofactors, such as ATP and NADPH, which are more plentiful during active photosynthesis (Hitchcock and Nichols, 1971). In agreement with previous studies on the developing primary leaves of barley, maize, rye grass and wheat (Bolton and Harwood, 1978), significantly higher levels of hexadecanoic and octadecanoic acid occurred in the leaf tip when compared to the base.

The higher levels of ascorbic and dehydroascorbic acid detected at the leaf tip in this study are consistent with their role as antioxidants that protect cells from damage resulting from the generation of free radicals, such as hydrogen peroxide, during photosynthesis (Foyer and Halliwell, 1976).

### 3.3 Impact of nitrate on metabolism

Appraisals of the extracted infrared peak areas confirmed that total protein, carbohydrate and lipid levels, detected in the leaf base of plants grown in the absence of nitrate were higher than levels seen in the tip. In contrast, levels in the leaf tip were higher in plants grown in the presence of nitrate, suggesting a switch in metabolism not detected by the physiological analyses.

Only glutamic acid, tyrosine and alanine levels increased commonly in all three leaf sections when plants grown in the presence of nitrate were compared to those grown in its absence, reflecting the different metabolic processes occurring in autotrophic and heterotrophic tissues. However, based upon comparisons between each respective leaf section, the numbers of metabolites that increased in the presence of nitrate are far greater. The availability of nitrate to the plant will lead to higher production of glutamic acid and glutamine via the GS/GOGAT pathway. As alanine is derived from glutamic acid, its level, as observed in this study, may also be expected to increase. This may also reflect the role of glutamine in plants as a nitrogen transport compound (Ireland, 1990; Tobin and Yamaya, 2001). The limited change of other amino acids might suggest amino acid synthesis in young leaves is not dependent on an external nitrogen source. Alternatively, amino acid flux through to other areas of metabolism, may limit changes detected by GC-TOF/MS measurements. Although technically much more demanding, fluxomics with mass isotopomer analysis (Winder et al., 2011) could be applied to specifically target amino acid flux (Gauthier et al., 2010).

In plants grown in the presence of nitrate, reduced levels of a number of sugar metabolites were observed in the basal section of the leaf when compared with levels in plants grown in the absence of nitrate. In contrast, maltose levels were higher in the leaf base of plants grown in the presence of nitrate. As leaf cells are respiring and not photosynthesising in the basal section, starch degradation is the most likely source of soluble and insoluble carbohydrate. It is well-established that starch breakdown leads to the production of maltose, and that a decline in starch accumulation occurs under nitrate stress (Niittylä et al., 2004; Weise et al., 2004), making it tempting to speculate that the higher level of maltose reflects an increased starch turnover.

An increased level of 2-oxoglutaric acid and succinic acid was seen in the leaf base of nitrate-treated plants. Scheible et al. (1997) demonstrated that nitrate supply to tobacco plants promoted the synthesis of 2-oxoglutaric acid and other TCA cycle organic acids by enhancing the transcript levels and enzyme activity of phospho*enol*pyruvate carboxylase, pyruvate kinase, citrate synthase, and isocitrate dehydrogenase. A GC–MS profiling study of tomato plants grown in the presence of nitrate also showed the levels of 2-oxoglutaric acid and other organic acids increased in the leaves (Urbanczyk-Wochniak and Fernie, 2005). As 2-oxoglutaric acid plays an important role in the TCA cycle and nitrogen assimilation (Stitt and Krapp, 1999), its metabolic shift between these two pathways must be tightly regulated. Glutamate dehydrogenase (GDH) catalyses a reversible enzymatic reaction involving the assimilation of ammonium into glutamic acid and the deamination of glutamic acid into 2-oxoglutaric acid and ammonium (Lancien et al., 2000). The direction of the GDH enzymatic reaction depends on the nitrogen and carbon source. Furthermore, GDH activity is very much under the control of NADH/NAD. The increased level of glutamic acid in plants grown in the presence of nitrate favours the deamination reaction of 2-oxoglutaric acid production (Lancien et al., 2000) in the base of nitrate-treated plants.

An increase in the level of malic acid at the tip of the leaf was observed when plants grown in the presence of nitrate were compared with plants grown in its absence. Malic acid concentrations are known to rise in response to surplus photosynthetic electron transport (Backhausen et al., 1998), especially during periods of nitrate assimilation (Schieble et al., 2000). In spinach and tobacco, nitrate reduction stimulates the anaplerotic production of malic acid to counter the imbalances in charge and pH caused by its assimilation (Muller et al., 2001; Schieble et al., 2000). As nitrate assimilation is dependent on substrate availability (Forde, 2002), and nitrate is more actively assimilated in the mature regions of wheat leaves (Tobin et al., 1988), the increase in malic acid levels at the leaf tip from nitrate-treated plants is not unexpected. Malic acid has an important role in the coordination of photosynthesis, glycolysis, TCA activity, glyoxysomal/peroxisomal activity and nitrate assimilation, as seen by its central positioning in the network correlation (Fig. S2e; Champigny, 1995; Hanning and Heldt, 1993; Martinoia and Rentsch, 1994; Muller et al., 2001; Schieble et al., 2000). The decreased malic acid level observed in the leaf base might be related to its degradation or conversion to other form(s) of metabolites in specific response processes to nitrate induction.

The level of trehalose was significantly higher in the primary leaves of plants grown in the absence of nitrate compared to those grown in its presence. A clear role of trehalose in stress tolerance, in particular drought, has been demonstrated for cryptobiotic species, such as the desiccation-tolerant *Selaginella lepidophylla* (Zentella et al., 1999), and higher vascular plants, like *Myrothamnus flabellifolius* (Bianchi et al., 1993; Drennan et al., 1993). Our results could reflect trehalose being produced by plants experiencing stress due to a lack of nitrate in the nutrient media. Trehalose accumulation in the leaf base may also protect the cells of the meristem. The inverse relationship between trehalose and G-6-P in plants grown in the presence of nitrate compared to those grown in its absence is indicative of sugar phosphates being diverted away from glycolysis into trehalose synthesis (Paul et al., 2008; Pellny et al., 2004; Schluepmann et al., 2003).

## 4 Conclusions

This study has shown that non-targeted metabolite profiling can detect changes along the developing wheat leaf of plants grown under different nitrate conditions and provides an insight into the metabolic adjustments that occur during leaf development. The clearest insight of plant metabolic differences during wheat leaf development was that obtained using PC–DFA for FT-IR data or multiblock C-PCA in the case of the GC-TOF/MS data. However, by combining the loadings derived from such multivariate analyses, with the results of univariate significance testing and BN analysis, a more detailed consensus interpretation and appreciation of the most important biological relationships and complexities between the metabolites and various experimental conditions is obtained. This study is one of the first to illustrate that BN analysis is a suitable approach for identifying significant metabolite differences which complements the commonly applied PCA and univariate significance tests. The various chemometric analyses revealed that different developmental stages along the primary wheat leaf could be distinguished from one another on the basis of their metabolite composition. Furthermore, growing plants in the presence or absence of nitrate had an additional impact on metabolite levels during wheat leaf development. The change in metabolites along the developing wheat leaf may be taken as indicative of different metabolic processes occurring within young and mature wheat leaf cells, including photosynthesis, respiration, nitrogen metabolism, sugar metabolism, fatty acid synthesis and the ascorbate–glutathione pathway. Our study also confirms that nitrate nutrition has an impact on leaf metabolism with high nitrate supply resulting in increases of some amino acids and organic acids and decreases in the level of several carbohydrates. As the data generated in this study match the interpretation from other metabolic studies, this work can now be incorporated into further functional studies to explore, for example, metabolite regulation during leaf development or to examine nitrogen use efficiency. Furthermore, combining this approach with the type of quantitative transcriptomic and proteomic analyses recently performed in developing maize leaves (Ponnala et al., 2014) will produce a powerful methodology for integrated metabolic modelling.

## 5 Experimental procedures

### 5.1 Plant growth and physiological analysis

For physiological studies wheat (*T. aestivum* L. cv Maris Huntsman) seeds from Plant Breeding International (Cambridge, UK) were grown in Levingtons M2 medium nutrient potting compost (Levington Horticulture Ltd., www.levington.com) in a controlled environment chamber (Fi-totron PG1400; Sanyo Gallenkamp, www.sanyo**-**biomedical.co.uk) with a 16 h photoperiod at 20 °C/10 °C and a constant humidity of 70%. Quantum flux was measured daily at 2 h into the light period with a Skye Light metre (Skye Instruments, www.skyeinstruments**.**com) and ranged between 232 and 348 μmol m^−2^ s^−1^ photosynthetic photon flux. For the metabolomics work, wheat (*T. aestivum* L. cv Paragon) seeds from Plant Breeding International were grown on 0.2% (w/v) Phytagel nutrient media (Paul and Stitt, 1993) in the presence or absence of 10 mM KNO_3_ as described by Gummadova et al. (2007), with all other growth conditions as described above.

Plants were harvested 2–3 h into the photoperiod after 7 or 8 days, when leaf height reached approximately 12 cm. The primary leaf was dissected from the seedling and 2 cm sections cut as described previously (Gummadova et al., 2007). All harvesting processes were carried out by hand, except for the metabolite profiling experiments where forceps were used to handle material. Following harvesting, tissue sections were either used immediately or flash frozen in liquid nitrogen and stored at −80 °C.

Mesophyll cell number was determined according to Dean and Leech (1982) with transverse sections taken along the leaf length of 5 primary wheat leaves at 5 mm intervals. Cell age was calculated by measuring the displacement velocity of marked regions along the leaf as described by Hopkins et al. (2002). The chlorophyll concentration was determined according to the method of Arnon (1949).

Photosynthesis was measured with a Hansatech leaf disc electrode (Hansatech Ltd., Norfolk, UK) as the rate of CO_2_ dependent O_2_ evolution in 1 cm transverse leaf sections from 10 primary leaves taken at 0, 20, 40, 60 and 80 mm above the leaf base, with saturating CO_2_ (5% v/v) and light (PAR at 900 μmol m^−2^ s^−1^) according to Walker (1990). Dark respiration was measured in the same way as photosynthesis but in the absence of light.

The total soluble and insoluble carbohydrate content of primary leaf tissue was determined colorimetrically at 623 nm using Dreywoods anthrone reagent (Morris, 1948). For determining amino acid free pools, sequential 1 cm tissue sections were taken from 5 primary leaves, frozen in liquid N_2_ and ground to a fine powder with 1 ml 80% (v/v) ethanol, and left to stand for 30 min at 4 °C. Following vortexing, the extract was centrifuged at 10,000*g* for 10 min and the resulting supernatant centrifuged a further 2 times under the same conditions. The pooled supernatant was frozen in liquid N_2_ and freeze dried overnight before re-dissolving in 1 ml of 12.5 μm l-α-aminobutyric acid (AABA) and centrifuging at 10,000*g* for 10 min. The supernatant was kept at 4 °C and centrifuged at 10,000*g* for 10 min immediately prior to HPLC analysis (LKB Bromma 2156 Solvent controller, 2152 LC Controller, 2159 HPLC pump) using a 3.9 × 150 mm Resolve C18 90A 5 μm reverse phase column (Waters Chromatography, www.waters.com) with a LDC Analytical-FluroMonitor III florescence detector (LDC Analytical Inc., Florida, USA).

### 5.2 Leaf sample processing for metabolite profiling

Freeze-dried primary leaf material from wheat grown on Phytagel media, corresponding to leaf tip (upper 2 cm), leaf base (lower 2 cm), and mid leaf (6–8 cm from base), was ground in 2 ml microcentrifuge tubes containing a clean 5 mm stainless steel ball bearing for 120 s at 25 cycles per second with an MM200 ball mill (Retsch, www.retsch.com). The grinding components of the mill were pre-cooled in liquid N_2_.

### 5.3 FT-IR preparation and analysis

Prior to sample loading, a ‘96 well’ silicon transmission plate (Bruker, www.bruker.com) was pre-washed in analytical grade methanol three times followed by dH_2_O three times, and the plate dried. To 30 mg (±1 mg) of ground leaf tissue 1.5 ml of sterile ultra pure dH_2_O was added and the sample thoroughly mixed. Thirty microlitre homogenates of each biological replicate were loaded onto the pre-washed sample plate to generate technical replicates, and three readings were taken from each sample spot to serve as analytical replicates. The plate was oven dried at 50 °C until samples were completely dry prior to loading into the motorised high-throughput stage (HTS-XT; Bruker) attached to a Bruker Equinox 55 FT-IR (Winder et al., 2004, 2006). The FT-IR transmission mode protocol was based precisely on the method previously described by Harrigan et al. (2004). Spectra were collected over the wavelength range of 4000–600 cm^−1^ with a resolution of 4 cm^−1^. To improve signal-to-noise ratio, the resulting spectra were co-added and averaged. Spectra were displayed in terms of absorbance as calculated using Opus 4 software, which uses the background spectrum of the reference well subtracted from the spectra recorded from the sample wells.

### 5.4 Extraction for GC-TOF/MS metabolite profiling

Homogenised leaf material was pre-weighed (50 ± 1 mg) into 2 ml microcentrifuge tubes (Eppendorf, UK. PN 0030 120.094). The metabolite extraction procedure used was based on that of Fiehn et al. (2000) and further developed by Lisec et al. (2006), and is previously described in detail in Biais et al. (2009). The internal standard solution consisted of 0.3 mg ml^−^^1^ succinic acid-*d_4_*, glycine-*d_5_* and malonic acid-*d_2_* dissolved in HPLC grade water. Once extracted, the samples were analysed within a month.

### 5.5 GC-TOF/MS analysis and data processing

Polar extracts were dried, derivatised and analysed by GC (Agilent 6890N gas chromatograph, Agilent Technologies Inc., www.agilent.com) coupled to an electron impact TOF/MS instrument (Pegasus III, LECO Corp., St. Joseph, USA; http://www.leco.com) following the method described in Biais et al. (2009) and Moing et al. (2011). Using ChromaTof v2.15, raw data processing (chromatographic deconvolution) was performed where S/N threshold was set at 10, baseline offset at 1.0, data points for averaging at 5, and peak width at 3. Metabolite peaks were identified by matching against three mass spectral libraries, NIST/EPA/NIH05 (http://www.nist.gov/srd/nist1.htm), the Golm Metabolome Database (GMDB; Hummel et al., 2010; Kopka et al., 2005; http://csbdb.mpimp-golm.mpg.de/csbdb/gmd/gmd.htm1), and an in-house mass spectral/RI library (Begley et al., 2009; Brown et al., 2009). Identifications followed MSI guidelines (Sumner et al., 2007) and were only considered as unambiguous (identification level 1) if a matching score of >700 was attained when comparing the sample mass spectrum with that of an authentic reference compound (Sigma–Aldrich or Acros Organics) analysed under the same conditions and instrument and showing the same RI (±10). Prior to further analysis, all of the deconvolved and aligned GC-TOF/MS profiles were exported to Microsoft Excel. Peak area data were corrected for derivatisation and sample injector errors using the succininc-*d_4_* acid internal standard, while sample weight error, which was relatively small (±2%), was not necessary to correct for. In addition the normalised peak areas for each metabolite were imported into MatLab R2008a (The MathWorks Inc., www.mathworks.com) where box and whisker plots were generated for the experimental group averages. Within the plots, the box represents the interquartile range (25% and 75%), the whiskers (error bars) represent data points not considered as outliers defined by 1.5× the interquartile range deviation from the mean. In cases where the data distribution is very tight and values lie only just outside of the 25% or 75%, the whiskers may not be clearly visible due to overlap with the box.

### 5.6 Principal Component–Discriminant Function Analysis of FT-IR metabolite fingerprints

The spectra obtained from FT-IR were converted to ASCII format from the instrument manufacturer’s software and imported into MatLab R2008a (The MathWorks Inc., www.mathworks.com). For FT-IR data, after Standard Normal Variate (SNV) baseline correction, the first derivative spectra were calculated using the Savitzky–Golay algorithm with 5-point smoothing (Savitzky and Golay, 1964). The data were first analysed using the unsupervised clustering method PCA (Jolliffe, 1986), which was followed by supervised PC-DFA (Manly, 1994). PCA and PC-DFA (Goodacre et al., 1998) were performed and validated in an identical manner as previously described (Allwood et al., 2006; Biais et al., 2009; Kaderbhai et al., 1995).

### 5.7 Multiblock Consensus-Principal Component Analysis of GC-TOF/MS metabolite profiles

The deconvolved and internal standard normalised GC-TOF/MS peak areas were directly imported from Microsoft Excel into MatLab R2008a (The MathWorks Inc., www.mathworks.com). Multiblock Consensus (C)-PCA was performed as described previously (Biais et al., 2009). The first C-PCA model arranged the data into two blocks consisting of nitrate supplemented and nitrate deprived samples. The second C-PCA model arranged the data into three blocks consisting of leaf base, mid leaf, and leaf tip, wheat leaf sections. After arrangement of data into blocks, with each experimental class consisting of a balanced number of sample replicates (6), each block was auto-scaled (i.e. each variable has a mean of 0 and a standard deviation of 1), and C-PCA was applied with results displayed as scores and loadings multiblock bi-plots. Additionally, a Wilcoxon rank-sum test was applied to test the difference between GC-TOF/MS profile variables at a 95% confidence limit (FDR *q*-value 0.05).

### 5.8 Bayesian network analysis

The BN analysis was undertaken based on the approach described by Correa and Goodacre (2011), only library-matched (identified) metabolite features of the basal and tip sections of the leaf were analysed, variables were only ranked as significant following the *t*-test after down adjustment for FDR correction (*q*-value 0.05). All statistical analyses followed recommendations from the metabolomics standards initiative (Goodacre et al., 2007b).

## Supplementary data

Supplementary Figure S1 Transmission electron micrograph of mesophyll cell development in wheat primary leaf tissue. Transverse sections of primary leaves (7 days old) were fixed in glutaraldehyde, embedded in Spurr’s resin, and ultra-thin sections taken at (a) 70, (b) 50, (c) 40, (d) 20, (e) 10 and (f) 5 mm above the basal meristem. Sections were double stained in uranyl acetate and lead citrate using standard procedures (Kuo, 2007) before being examined on a Philips 301 Transmission Electron Microscope. Abbreviations a = air space, c = cytosol, cp = chloroplast, cw = cell wall, m = mitochondria, n = nucleus, v = vacuole. Bars = 2 μm.

Supplementary Figure S2 Bayesian network (BN) analysis presented in the traditional network topology. Bayesian network (BN) analysis was performed to search for strong probabilistic correlations between metabolites with respect to growth conditions and leaf position. This figure presents the BN analysis results in the more traditional network topology. Correlations (the values are given on the edges of the graph) were searched under four scenarios: The first searched for strong correlations between metabolites within the base and tip of the leaf when grown in the absence of nitrate with the networks displayed with respect to (a) tip N− treatment and (b) base N− treatment correlations. The second searched for strong correlations between metabolites within the base and tip of the leaf when grown in the presence of nitrate with the networks displayed with respect to (c) tip N+ treatment and (d) base N+ treatment. The third searched for strong correlations between metabolites within the leaf tip when grown in the absence and presence of nitrate with respect to (e) N+ condition and (f) N− condition. Finally the fourth searched for strong correlations between metabolites within the base leaf when grown in the absence and presence of nitrate with respect to (g) N+ condition and (h) N− condition.

Supplementary Figure S3 Bayesian network (BN) analysis presented as plots of peak area log_2_ fold change. Bayesian network (BN) analysis was performed to search for strong correlations between metabolites with respect to growth conditions and leaf position. This figure presents the BN analysis results in the form of plots of peak area log_2_ ratios, thus revealing the regulation of each metabolite with respect to leaf position or nitrate treatment. (a) Leaf tip metabolites elevated in contrast to leaf base under nitrate − condition [−log_2_ (Base N−/Tip N−)], (b) leaf base metabolites elevated in contrast to leaf tip under nitrate − condition [log_2_ (Base N−/Tip N−)], (c) leaf tip metabolites elevated in contrast to leaf base under nitrate + condition [−log_2_ (Base N+/Tip N+)], (d) leaf base metabolites elevated in contrast to leaf tip under nitrate + condition [log_2_ (Base N+/Tip N+)], (e) leaf tip metabolites elevated under nitrate + condition [Tip − log_2_ (N−/N+)], (f) leaf tip metabolites elevated under nitrate − condition [Tip log_2_ (N−/N+)], (g) leaf base metabolites elevated under nitrate + condition [Base − log_2_ (N−/N+)], and (h) leaf base metabolites elevated under nitrate − condition [Base log_2_ (N−/N+)].

Supplementary Table S1 Metabolites that show significant changes in their levels during the development of the primary wheat leaf in plants grown in the presence or absence of nitrate (metabolite identifications, class averages and standard errors, results of Wilcoxon rank-sum test (FDR *q*-value 0.05) and Bayesian networking *t*-test (FDR *q*-value 0.05).
