# Supplementary figures and images for: Profiling of spatial metabolite distributions in wheat leaves under normal and nitrate limiting conditions
Source: Phytochemistry. 2015 Jul;115:99–111. doi: 10.1016/j.phytochem.2015.01.007 (PMC4518043; doi:10.1016/j.phytochem.2015.01.007)

Figure S1

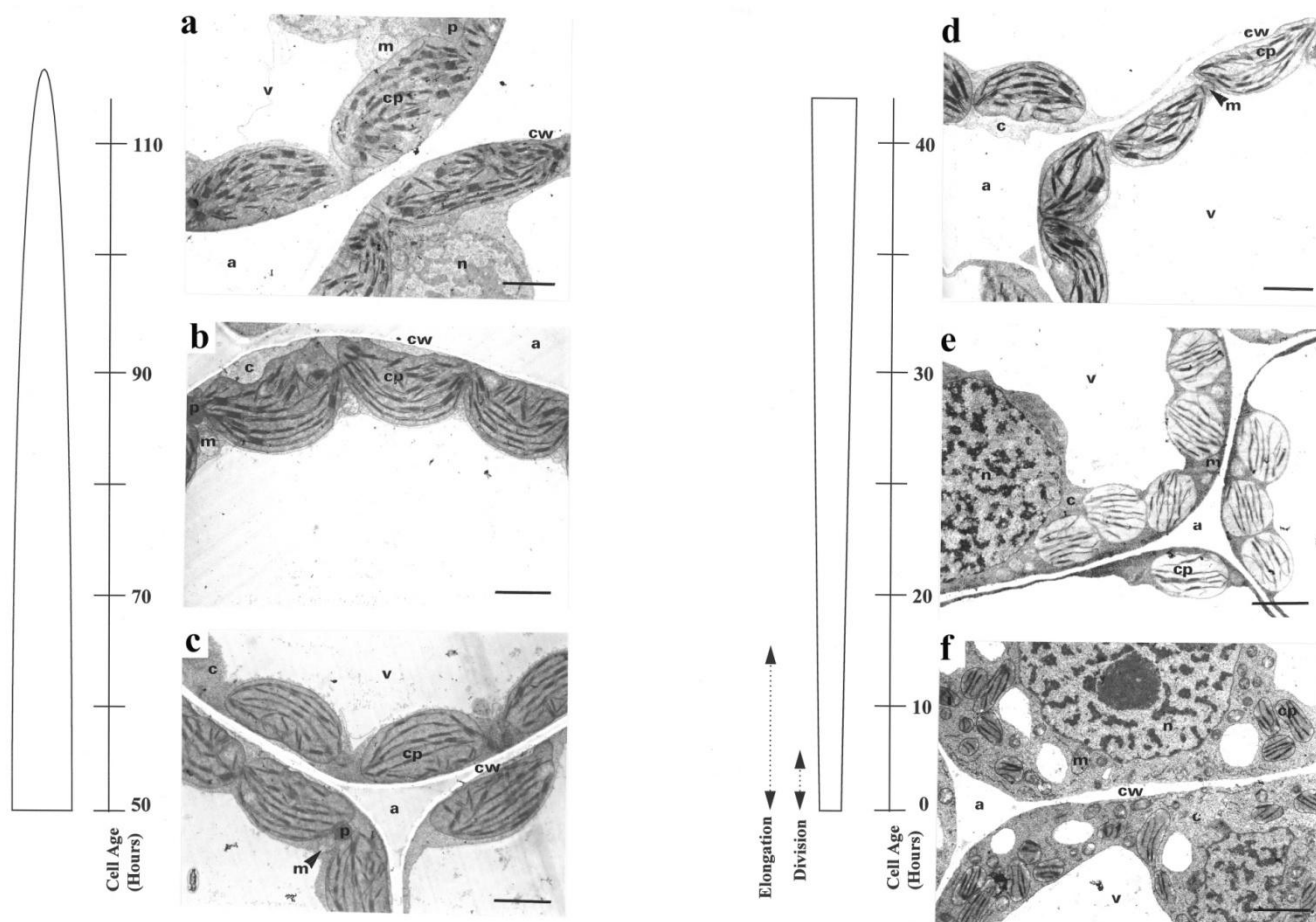

Supplement: Supplementary Figure S1 — Transmission electron micrograph of mesophyll cell development in wheat primary leaf tissue. Transverse sections of primary leaves (7 days old) were fixed in glutaraldehyde, embedded in Spurr’s resin, and ultra-thin sections taken at (a) 70, (b) 50, (c) 40, (d) 20, (e) 10 and (f) 5 mm above the basal meristem. Sections were double stained in uranyl acetate and lead citrate using standard procedures (Kuo, 2007) before being examined on a Philips 301 Transmission Electron Microscope. Abbreviations a = air space, c = cytosol, cp = chloroplast, cw = cell wall, m = mitochondria, n = nucleus, v = vacuole. Bars = 2 μm. [file mmc1.pdf]

Figure S2

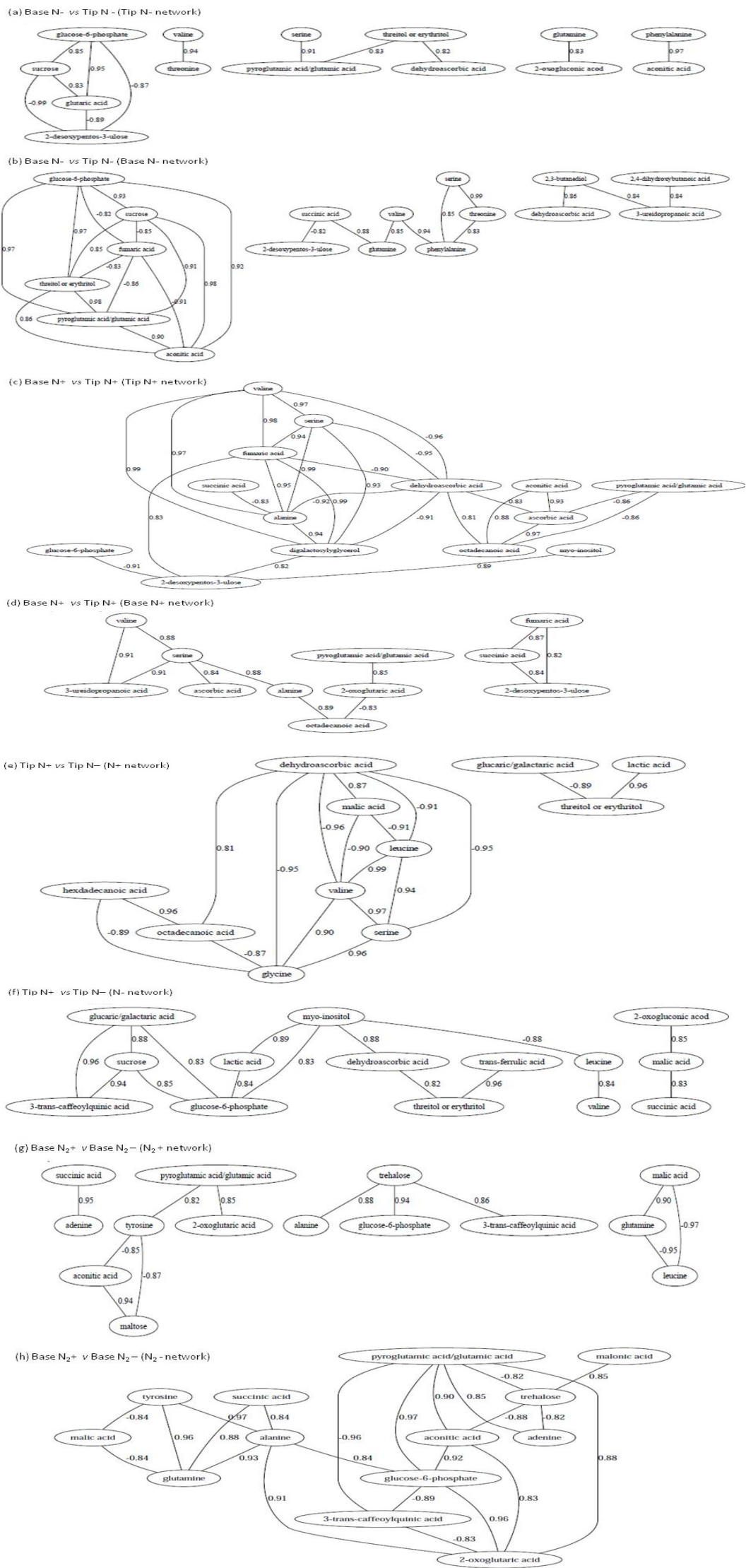

Supplement: Supplementary Figure S2 — Bayesian network (BN) analysis presented in the traditional network topology. Bayesian network (BN) analysis was performed to search for strong probabilistic correlations between metabolites with respect to growth conditions and leaf position. This figure presents the BN analysis results in the more traditional network topology. Correlations (the values are given on the edges of the graph) were searched under four scenarios: The first searched for strong correlations between metabolites within the base and tip of the leaf when grown in the absence of nitrate with the networks displayed with respect to (a) tip N− treatment and (b) base N− treatment correlations. The second searched for strong correlations between metabolites within the base and tip of the leaf when grown in the presence of nitrate with the networks displayed with respect to (c) tip N+ treatment and (d) base N+ treatment. The third searched for strong correlations between metabolites within the leaf tip when grown in the absence and presence of nitrate with respect to (e) N+ condition and (f) N− condition. Finally the fourth searched for strong correlations between metabolites within the base leaf when grown in the absence and presence of nitrate with respect to (g) N+ condition and (h) N− condition. [file mmc2.pdf]
