# Supplementary material for: Profiling of spatial metabolite distributions in wheat leaves under normal and nitrate limiting conditions
Source: Phytochemistry. 2015 Jul;115:99–111. doi: 10.1016/j.phytochem.2015.01.007 (PMC4518043; doi:10.1016/j.phytochem.2015.01.007)
Supplement: Supplementary Figure S3 — Bayesian network (BN) analysis presented as plots of peak area log2 fold change. Bayesian network (BN) analysis was performed to search for strong correlations between metabolites with respect to growth conditions and leaf position. This figure presents the BN analysis results in the form of plots of peak area log2 ratios, thus revealing the regulation of each metabolite with respect to leaf position or nitrate treatment. (a) Leaf tip metabolites elevated in contrast to leaf base under nitrate − condition [−log2 (Base N−/Tip N−)], (b) leaf base metabolites elevated in contrast to leaf tip under nitrate − condition [log2 (Base N−/Tip N−)], (c) leaf tip metabolites elevated in contrast to leaf base under nitrate + condition [−log2 (Base N+/Tip N+)], (d) leaf base metabolites elevated in contrast to leaf tip under nitrate + condition [log2 (Base N+/Tip N+)], (e) leaf tip metabolites elevated under nitrate + condition [Tip − log2 (N−/N+)], (f) leaf tip metabolites elevated under nitrate − condition [Tip log2 (N−/N+)], (g) leaf base metabolites elevated under nitrate + condition [Base − log2 (N−/N+)], and (h) leaf base metabolites elevated under nitrate − condition [Base log2 (N−/N+)]. [file mmc3.pdf]

Figure S3

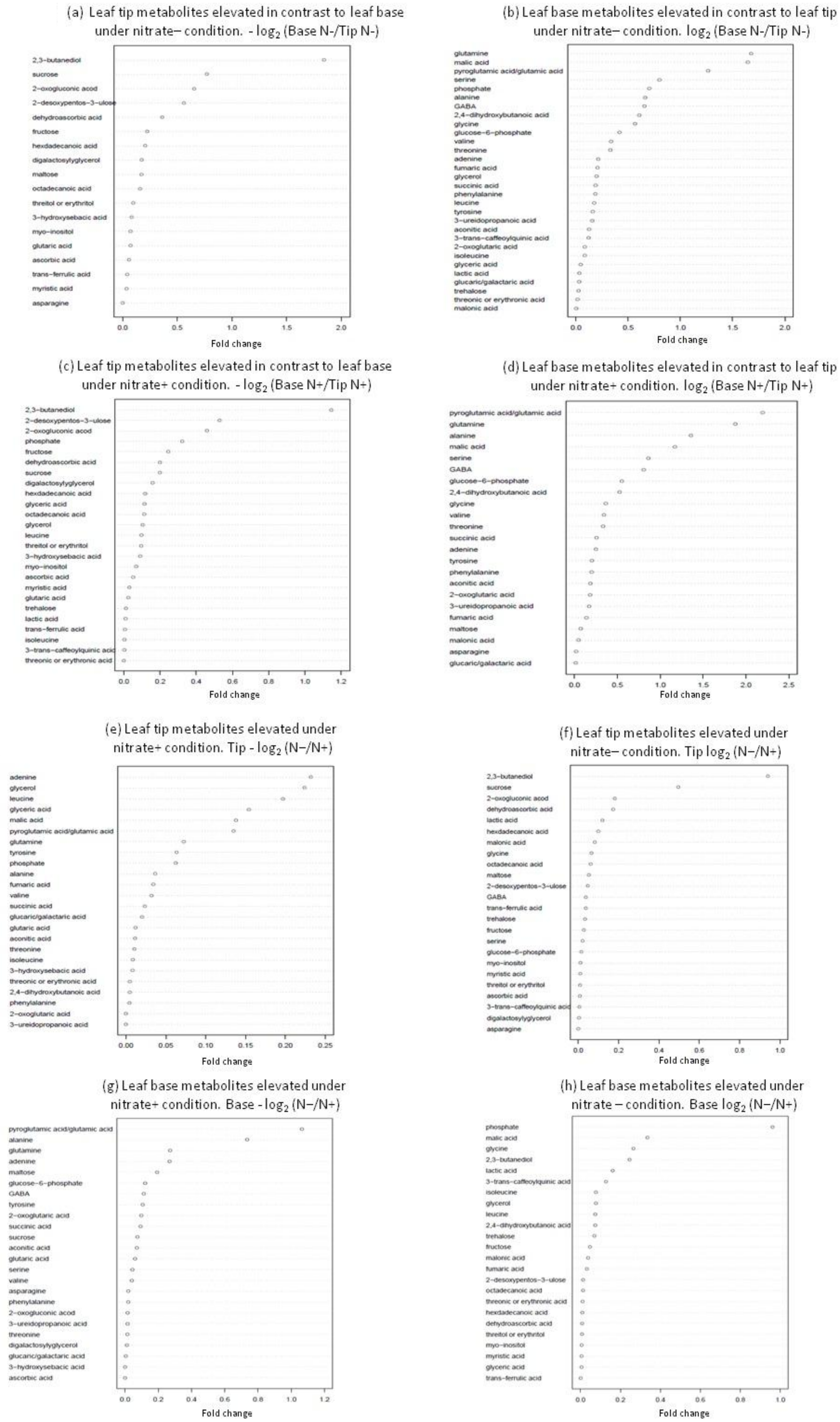
(a) Leaf tip metabolites elevated in contrast to leaf base under nitrate– condition. - log2 (Base N-/Tip N-)
2,3–butanediol
sucrose
2–oxogluconic acod
2–desoxypentos–3–ulose
dehydroascorbic acid
fructose
hexdadecanoic acid
digalactosylyglycerol
maltose
octadecanoic acid
threitol or erythritol
3–hydroxysebacic acid
myo–inositol
glutaric acid
ascorbic acid
trans–ferrulic acid
myristic acid
asparagine
0.0 0.5 1.0 1.5 2.0
Fold change
(b) Leaf base metabolites elevated in contrast to leaf tip under nitrate– condition. log2 (Base N-/Tip N-)
glutamine
malic acid
pyroglutamic acid/glutamic acid
serine
phosphate
alanine
GABA
2,4–dihydroxybutanoic acid
glycine
glucose–6–phosphate
valine
threonine
adenine
fumaric acid
glycerol
succinic acid
phenylalanine
leucine
tyrosine
3–ureidopropanoic acid
aconitic acid
3–trans–caffeoylquinic acid
2–oxoglutaric acid
isoleucine
glyceric acid
lactic acid
glucaric/galactaric acid
trehalose
threonic or erythronic acid
malonic acid
0.0 0.5 1.0 1.5 2.0
Fold change
(c) Leaf tip metabolites elevated in contrast to leaf base under nitrate+ condition. - log2 (Base N+/Tip N+)
2,3–butanediol
2–desoxypentos–3–ulose
2–oxogluconic acod
phosphate
fructose
dehydroascorbic acid
sucrose
digalactosylyglycerol
hexadecanoic acid
glyceric acid
octadecanoic acid
glycerol
leucine
threitol or erythritol
3–hydroxysebacic acid
myo–inositol
ascorbic acid
myristic acid
glutaric acid
trehalose
lactic acid
trans–ferrulic acid
isoleucine
3–trans–caffeoylquinic acid
threonic or erythronic acid
0.0 0.2 0.4 0.6 0.8 1.0 1.2
Fold change
(d) Leaf base metabolites elevated in contrast to leaf tip under nitrate+ condition. log2 (Base N+/Tip N+)
pyroglutamic acid/glutamic acid
glutamine
alanine
malic acid
serine
GABA
glucose–6–phosphate
2,4–dihydroxybutanoic acid
glycine
valine
threonine
succinic acid
adenine
tyrosine
phenylalanine
aconitic acid
2–oxoglutaric acid
3–ureidopropanoic acid
fumaric acid
maltose
malonic acid
asparagine
glucaric/galactaric acid
0.0 0.5 1.0 1.5 2.0 2.5
Fold change
(e) Leaf tip metabolites elevated under nitrate+ condition. Tip - log2 (N–/N+)
adenine
glycerol
leucine
glyceric acid
malic acid
pyroglutamic acid/glutamic acid
glutamine
tyrosine
phosphate
alanine
fumaric acid
valine
succinic acid
glucaric/galactaric acid
glutaric acid
aconitic acid
threonine
isoleucine
3–hydroxysebacic acid
threonic or erythronic acid
2,4–dihydroxybutanoic acid
phenylalanine
2–oxoglutaric acid
3–ureidopropanoic acid
0.00 0.05 0.10 0.15 0.20 0.25
Fold change
(f) Leaf tip metabolites elevated under nitrate– condition. Tip log2 (N–/N+)
2,3–butanediol
sucrose
2–oxogluconic acod
dehydroascorbic acid
lactic acid
hexadecanoic acid
malonic acid
glycine
octadecanoic acid
maltose
2–desoxypentos–3–ulose
GABA
trans–ferrulic acid
trehalose
fructose
serine
glucose–6–phosphate
myo–inositol
myristic acid
threitol or erythritol
ascorbic acid
3–trans–caffeoylquinic acid
digalactosylyglycerol
asparagine
0.0 0.2 0.4 0.6 0.8 1.0
Fold change
(g) Leaf base metabolites elevated under nitrate+ condition. Base - log2 (N–/N+)
pyroglutamic acid/glutamic acid
alanine
glutamine
adenine
maltose
glucose–6–phosphate
GABA
tyrosine
2–oxoglutaric acid
succinic acid
sucrose
aconitic acid
glutaric acid
serine
valine
asparagine
phenylalanine
2–oxogluconic acod
3–ureidopropanoic acid
threonine
digalactosylyglycerol
glucaric/galactaric acid
3–hydroxysebacic acid
ascorbic acid
0.0 0.2 0.4 0.6 0.8 1.0 1.2
Fold change
(h) Leaf base metabolites elevated under nitrate – condition. Base log2 (N–/N+)
phosphate
malic acid
glycine
2,3–butanediol
lactic acid
3–trans–caffeoylquinic acid
isoleucine
glycerol
leucine
2,4–dihydroxybutanoic acid
trehalose
fructose
malonic acid
fumaric acid
2–desoxypentos–3–ulose
octadecanoic acid
threonic or erythronic acid
hexadecanoic acid
dehydroascorbic acid
threitol or erythritol
myo–inositol
myristic acid
glyceric acid
trans–ferrulic acid
0.0 0.2 0.4 0.6 0.8 1.0
Fold change
